# The clinical outcomes of haploidentical stem cell transplantation (haplo-HSCT) for patients with therapy-related myelodysplastic syndrome: comparable to de novo myelodysplastic syndrome

**DOI:** 10.1007/s10238-023-01287-8

**Published:** 2024-02-08

**Authors:** Feifei Tang, Yunqi Wang, Yu Wang, Jian Jin, Wei Han, Yuhong Chen, Chenhua Yan, Lanping Xu, Xiaohui Zhang, Xiaojun Huang

**Affiliations:** 1 Peking University People’s Hospital, Peking University Institute of Hematology, National Clinical Research Center for Hematologic Disease, Beijing Key Laboratory of Hematopoietic Stem Cell Transplantation, Beijing, China; 2 Peking-Tsinghua Center for Life Sciences, Beijing, China

**Keywords:** Myelodysplastic syndrome, Therapy-related myelodysplastic syndrome, Haploidentical stem cell transplantation, haploidentical

## Abstract

Therapy-related myelodysplastic syndrome (t-MDS) is defined as a complication in patients with cancer following exposure to chemotherapy and/or radiotherapy and has an inferior outcome compared with de novo myelodysplastic syndrome (de novo MDS). This study aimed to estimate and compare the clinical outcomes of haploidentical stem cell transplantation (haplo-HSCT) for t-MDS and de novo MDS. We retrospectively analyzed 96 patients with MDS who received haplo-HSCT between January 2015 and December 2021. Eleven patients with t-MDS and 85 patients with de novo MDS were matched using the case-pair method in a 1:8 ratio with the following pairing criteria: (1) sex, (2) age (± 5 years), (3) year of haplo-HSCT (± 2 years), and (4) blast cell counts (≥ 5% or not). The 3-year overall survival and disease-free survival after haplo-HSCT for t-MDS versus de novo MDS patients were 72.7% versus 75.1% (*P* = 0.99) and 54.5% versus 67.0% (*P* = 0.50), respectively. The 3-year cumulative incidence of relapse was 36.4% versus 15.5% (*P* = 0.08), respectively. In multivariate analysis, there was no difference in relapse between t-MDS and de novo MDS. The 3-year cumulative non-relapse mortality rates were 9.1% versus 17.6% (*P* = 0.45), respectively. This study confirmed the comparable clinical outcomes of haplo-HSCT on the prognosis of t-MDS and de novo MDS.

## Introduction

Myelodysplastic syndromes (MDS) are a group of myeloid neoplasms characterized by bone marrow dysplasia, ineffective hematopoiesis, peripheral blood cytopenia, and a high-risk transformation to acute myeloid leukemia (AML) [1–3]. De novo MDS and t-MDS are subtypes of MDS with different biology and prognosis. t-MDS is defined as a complication of patients with cancer following exposure to chemotherapy and/or radiotherapy and has an inferior outcome compared with de novo MDS, with a median survival of one year or less [4–8]. According to the 2022 WHO classification [9], myeloid neoplasms post-cytotoxic tumor (MN-pCT) replace the previous diagnosis called therapy-related myeloid neoplasms (t-MN), including both t-AML and t-MDS [10]. According to the 2022 European LeukemiaNet recommendations for AML, therapy-related factors, including radiotherapy, chemotherapy, and immune interventions and secondary AML that progressed from MDS and MDS/MPN (specify type), are adverse prognostic factors [11]. At present, MDS is merely classified and diagnosed according to morphological, flow cytometry, cytogenetic and molecular factors. Although t-MDS has not been regarded as a poor prognostic factor in various MDS classifications, it should also be taken into consideration.

Allogeneic hematopoietic cell transplantation (allo-HSCT) remains the only curative therapy strategy for t-MDS to date [5, 8, 12, 13]. Haploidentical stem cell transplantation (haplo-HSCT) provides an attractive treatment option for patients lacking an available identical sibling donor (ISD) who need urgent transplantation [14, 15]. There have been considerable advances in the field of haplo-HSCT, such as donor selection, conditioning regimens, and graft-versus-host disease prophylaxis, which have successfully improved the clinical outcomes of patients receiving haplo-HSCT. Our previous studies demonstrated comparable efficacy between haploidentical hematopoietic cell transplantation (haplo-HSCT) and identical sibling transplant for MDS [16]. However, prior studies on t-MDS did not separate haplo-HSCT from other types of allo-HSCT [4, 6, 17, 18]. Therefore, in this study, we compared and analyzed the clinical outcomes of haplo-HSCT for t-MDS with those of de novo MDS.

## Materials and methods

### Patients

We retrospectively reviewed the data of MDS patients who received haplo-HSCT at the Institute of Hematology, Peking University, Beijing, China, between January 2015 and December 31, 2021. We identified 11 t-MDS patients who received haplo-HSCT and used the case-pair method to match the control subjects in a 1:8 ratio with the following pairing criteria: (1) sex, (2) age (± 5 years), (3) year of haplo-HSCT (± 2 years), and (4) blast cell counts (≥ 5% or not). This matching method strictly controlled the confounding effect of time in the analysis process. Eighty-five patients with de novo MDS who received haplo-HSCT were finally matched as a set of control cases owing to the fact that 2 patients in t-MDS group did not achieve a 1:8 pairing. Eventually, 96 patients were eligible as subjects for this study. Informed consent was obtained from all patients for being included in the study. All methods were carried out in accordance with the Declaration of Helsinki, and the protocol was approved by the ethics committee of Peking University People's Hospital. Cytogenetic risk was defined based on the revised International Prognostic Scoring System (IPSS-R) criteria [19].

### Transplant procedure

All patients received myeloablative conditioning (MAC) regimen. The uniform conditioning regimen for haploidentical donors (HID) was cytarabine (4 g/m^2^ per day, intravenous infusion, days − 10 to − 9), busulfan (3.2 mg/kg per day, intravenous infusion, days − 8 to − 6), cyclophosphamide (1.8 g/m^2^ per day, intravenous infusion, days − 5 to − 4), semustine (250 mg/m^2^ per day, oral administration, day − 3), and rabbit antithymocyte globulin (ATG) (2.5 mg/kg per day, intravenous infusion, days − 5 to − 2) [20]. Granulocyte colony stimulating factor (G-CSF) (5 μg/kg) mobilizing bone marrow and/or peripheral blood grafts were infused on the day of collection for HSCT. GVHD prophylaxis consisted of cyclosporine (2.5 mg/kg, intravenous infusion, since day − 9), mycophenolate mofetil (1.0 g, oral administration, since day − 9), and short-term methotrexate (15 mg/m^2^, intravenous infusion, on day 1 and 10 mg/m^2^ on days 3, 5, and 11) for all patients. Bone marrow samples were evaluated at 1, 2, 3, 4.5, 6, 9, and 12 months after haplo-HSCT, and at every 6 months after thereafter, to monitor minimum residual disease (MRD) after haplo-HSCT [21].

### Definitions

The endpoints of our study were overall survival (OS) and disease-free survival (DFS), while the secondary observation targets included non-relapse mortality (NRM), the incidence of relapse and the incidences of acute GVHD (aGVHD), chronic GVHD (cGVHD) and influencing factors for clinical outcomes. OS time was defined as the time from transplantation to death from any cause or the last follow-up in surviving patients. DFS was defined as the time from transplantation to relapse or death from any cause. Relapse was defined as morphological evidence of the disease discovered in texting samples from the peripheral blood, bone marrow, or extramedullary sites or else by the recurrence and sustained presence of pre-transplantation chromosomal abnormalities [14]. NRM was defined as the time from transplantation to death from any cause without relapse. All data were calculated from the day of graft infusion. Neutrophil engraftment was defined as an absolute neutrophil count ≥ 0.5 × 10^9^/L on three consecutive days. Platelet engraftment was defined as no transfusion for 7 consecutive days and platelet count ≥ 20 × 10^9^/L. aGVHD and cGVHD were defined according to published criteria [22, 23].

### Statistical analysis

The endpoint of the last follow-up for all survivors was April 30, 2023. The median follow-up time of survivors was 34.9 months (range, 17.8–100.1 months). Baseline patient, disease, and transplant-related characteristics were analyzed between patients with de novo MDS and those with t-MDS by using the χ^2^ test and Fisher’s exact test for categorical variables and the Mann–Whitney U test for continuous variables. OS and DFS were calculated by using the Kaplan–Meier estimator with the log-rank test. The cumulative incidence of GVHD, relapse and NRM were calculated by using competing risk analysis. For GVHD, death from any cause was a competing event. For NRM, relapse was a competing event, and for relapse, NRM was a competing event. Multivariable analyses were performed using Cox proportional hazards regression to identify independent risk factors for NRM, relapse, DFS, and OS. The variables in the Cox proportional hazards regression model were as follows: patient age (< 35 years vs. ≥ 35 years), interval between MDS diagnosis and haplo-HSCT (interval < 30 months vs. interval ≥ 30 months), cytogenetic risk (high cytogenetic risk vs. non-high cytogenetic risk), graft type (BM + PB vs. PB), platelet engraftment (yes vs. no), treatment for t-MDS/de novo MDS prior to haplo-HSCT (chemotherapy vs. supportive care only) and MDS subtype (de novo MDS vs. t-MDS). All variables with *P* < 0.1 in the univariate analysis were allowed to be analyzed in the multivariate regression. The MDS subtype (de novo MDS vs. t-MDS) was forced into the Cox proportional hazards model regardless of the *P* value. Two-sided *P* values < 0.05 were considered statistically significant. Data analyses were calculated by using the Statistical Package for the Social Sciences (SPSS) software (SPSS Inc., Chicago, IL, USA), whereas competing risk analysis was performed using R software (version 3.2.1; http://www.r-project.org).

## Results

### Patient characteristics

Table 1 summarizes the characteristics of the 96 patients enrolled in this study. Among them, 11 (11.5%) were diagnosed with t-MDS, and 85 (88.5%) had de novo MDS. Two patients in t-MDS group did not achieve a 1:8 pairing. The median age of all patients was 48 years (range, 2–62 years) and the median age in t-MDS group and de novo MDS group was 51 years (range, 6–58 years) and 48 years (range, 2–62 years), respectively (*P* = 0.83). The median interval from MDS diagnosis to haplo-HSCT was 6 months (1–192 months). A higher proportion of t-MDS patients was found with the high-risk cytogenetic abnormalities compared to de novo MDS (45.5% vs. 18.8%, *P* = 0.04). Treatment for t-MDS prior to haplo-HSCT included supportive treatment (*n* = 5, 45.5%) and chemotherapy (*n* = 6, 54.5%).

**Table 1:** Baseline patient, disease, and transplant-related characteristics

Characteristics	De novo MDS (*n* = 85)	t-MDS (*n* = 11)	*P* value
Median age of patients, years (range)	48 (2–62)	51 (6–58)	0.83
< 50	45 (52.9)	5 (45.5)	
≥ 50	40 (47.1)	6 (54.5)	
Sex			0.93
Male	32 (37.6)	4 (36.4)	
Female	53 (62.4)	7 (63.6)	
WHO classification			0.59
MDS-MLD	2(2.4)	1 (9.1)	
MDS-RAEB-I	22 (25.9)	2 (18.2)	
MDS-RAEB-II	59(69.4)	8(72.7)	
MDS-RCC	2(2.4)	0	
Cytogenetic risk categories			0.04
Low risk	43 (50.6)	2 (18.2)	
Middle risk	26 (30.6)	4 (36.4)	
High risk	16 (18.8)	5 (45.5)	
IPSS-R			0.69
Very low	0	0	
Low	4 (4.7)	0	
Intermediate	14 (16.5)	1 (9.1)	
High	27 (31.8)	3 (27.3)	
Very high	40 (47.1)	7 (63.6)	
Blast cell, %	9.0% (0.0–19.0%)	15.0% (3.5–22.0%)	0.14
WBC in PB, × 10^9^/L (range)	2.8 (0.4–16.6)	2.9 (1.5–8)	0.90
Hb in PB, g/L(range)	74 (35–129)	70 (49–128)	0.85
PLT in PB, × 10^9^/L (range)	46 (4–324)	41 (17–179)	0.69
Treatment for t-MDS/de novo MDS prior to haplo-HSCT			0.04
Supportive care only	54 (63.5)	5 (45.5)	
Low-intensity chemotherapy	13 (15.3)	0	
Intensive chemotherapy	18 (21.2)	6 (54.5)	
Year of haplo-HSCT, median	2019 (2015–2021)	2020 (2016–2021)	0.96
Duration from diagnosis to haplo-HSCT, months (range)	6 (1–192)	5 (2–24)	0.53
Median mononuclear cells, × 10^8^/kg(range)	8.5 (4.3–23.8)	9 (7.1–11.3)	0.81
Median CD34 + counts, × 10^6^/kg(range)	2.8 (0–14.3)	3.6 (1.4–6.9)	0.19
Donor-patient sex match			0.52
Male–male	22 (25.9)	4 (36.4)	
Male–female	31 (36.5)	5 (45.5)	
Female–male	12 (14.1)	0	
Female–female	20 (23.5)	2 (18.2)	
Donor relationship			0.46
Father	20 (23.5)	3 (27.3)	
Mother	5 (5.9)	0	
Sibling	16 (18.8)	4 (36.4)	
Child	44 (51.8)	4 (36.4)	
Donor sex			0.2
Male	53 (62.4)	9 (81.8)	
Female	32 (37.6)	2 (18.2)	
Graft type			0.09
BM + PB	60 (70.6)	5 (45.5)	
PB	25 (29.4)	6 (54.5)	
Primary diagnosis			
Hematological		6 (54.5)	
Lung cancer		2 (18.2)	
Genital system carcinoma		2 (18.2)	
Thyroid carcinoma		1 (9.1)	
Months from primary diagnosis to MDS		57(22.7–120)	

*BM* Bone marrow, *haplo-HSCT* Haploidentical hematopoietic stem cell transplantation, *Hb* Hemoglobin, *IPSS-R* Revised International prognostic scoring system, *PB* Peripheral blood, *PLT* Platelet, *t-MDS* Therapy-related myelodysplastic syndrome, *WBC* White blood cell

Among the 11 patients with t-MDS, 6 (54.5%) patients had prior hematological malignancy, including AML (*n* = 3), chronic myeloid leukemia (*n* = 1), acute lymphoblastic leukemia (*n* = 1), and non-Hodgkin lymphoma (*n* = 1), and 5 (45.5%) patients had a history of solid malignancy, including 2 (18.2%) with lung cancer, 2 (18.2%) with genital system carcinoma, and 1 (9.1%) with thyroid carcinoma. The median latency time from the diagnosis of primary cancer and t-MDS was 57 months (22.7–120 months). None of the patients received radiotherapy. The median cycle of chemotherapy was 7 (range, 5–28 cycles). The chemotherapy drugs principally included alkylating agents, topoisomerase, and antimetabolic drugs. All t-MDS patients with solid malignancy underwent surgical resection. The primary cancer of all patients with t-MDS was cured at the time of haplo-HSCT. The median interval from t-MDS diagnosis to haplo-HSCT was 5 months (2–24 months).

### Engraftment and GVHD

All patients showed complete donor chimerism after transplantation. Incomplete neutrophil recovery was observed in 2 patients with de novo MDS. The median neutrophil engraftment times in the t-MDS and control cohort were 13 days (range, 11–20 days) and 12 days (range, 9–26 days), respectively (*P* = 0.35). Eight patients with de novo MDS failed to achieve platelet engraftment. The median platelet engraftment times of patients in the t-MDS and control cohort were 14 days (range, 9–102 days) and 15 days (range, 9–119 days), respectively (*P* = 0.84).

**Fig. 1:**
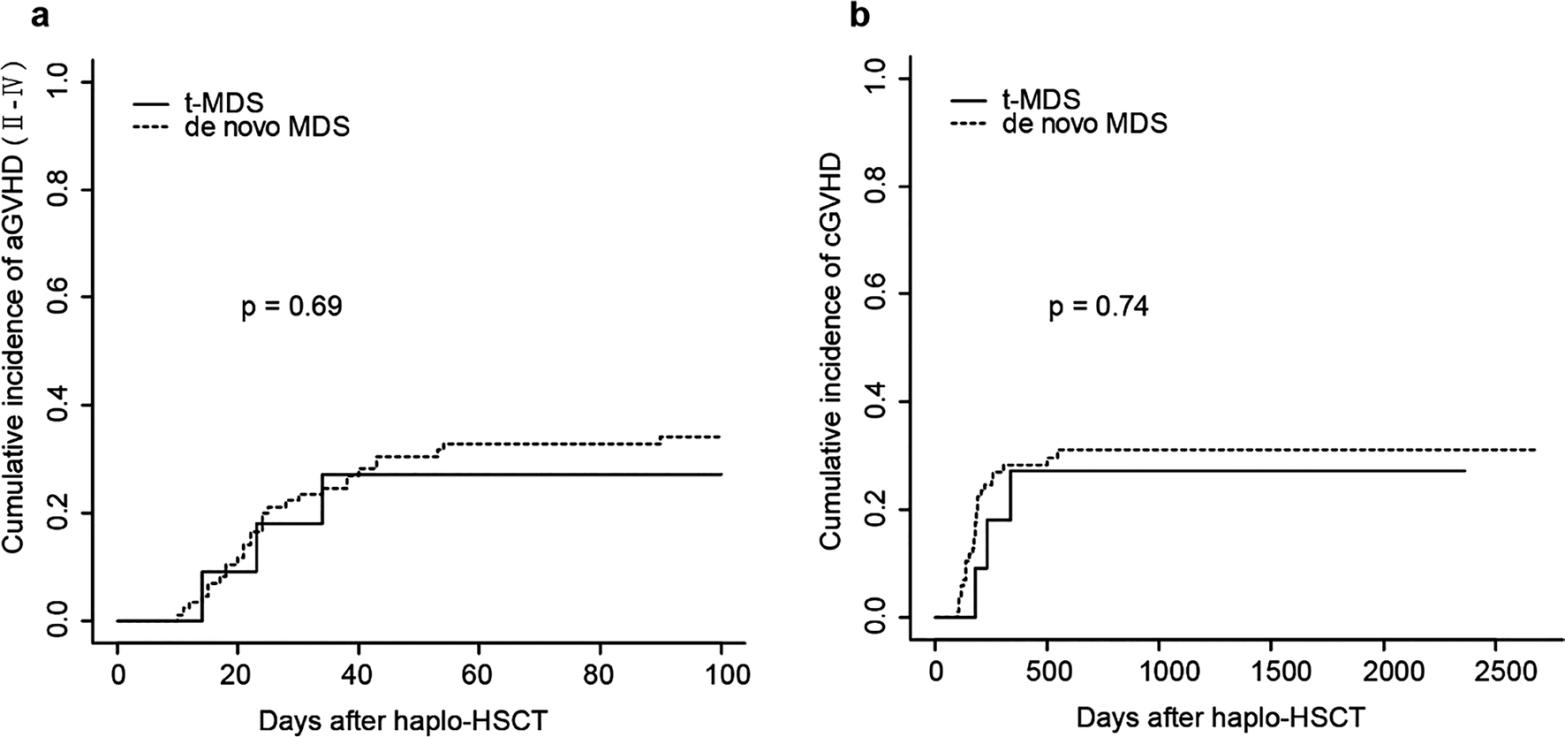
Cumulative incidence of aGVHD (II–IV) (**a**) and cGVHD (**b**) after haplo-HSCT in patients with t-MDS or de novo MDS

On 100 days after transplantation, the cumulative incidences of grade II–IV aGVHD in the t-MDS and control cohort were 27.3% and 32.9%, respectively (*P* = 0.69; Fig. 1a). The cumulative incidence of all-grade cGVHD in the t-MDS and control cohort were 27.3% and 31.2%, respectively (*P* = 0.74; Fig. 1b).

### Relapse

**Fig. 2:**
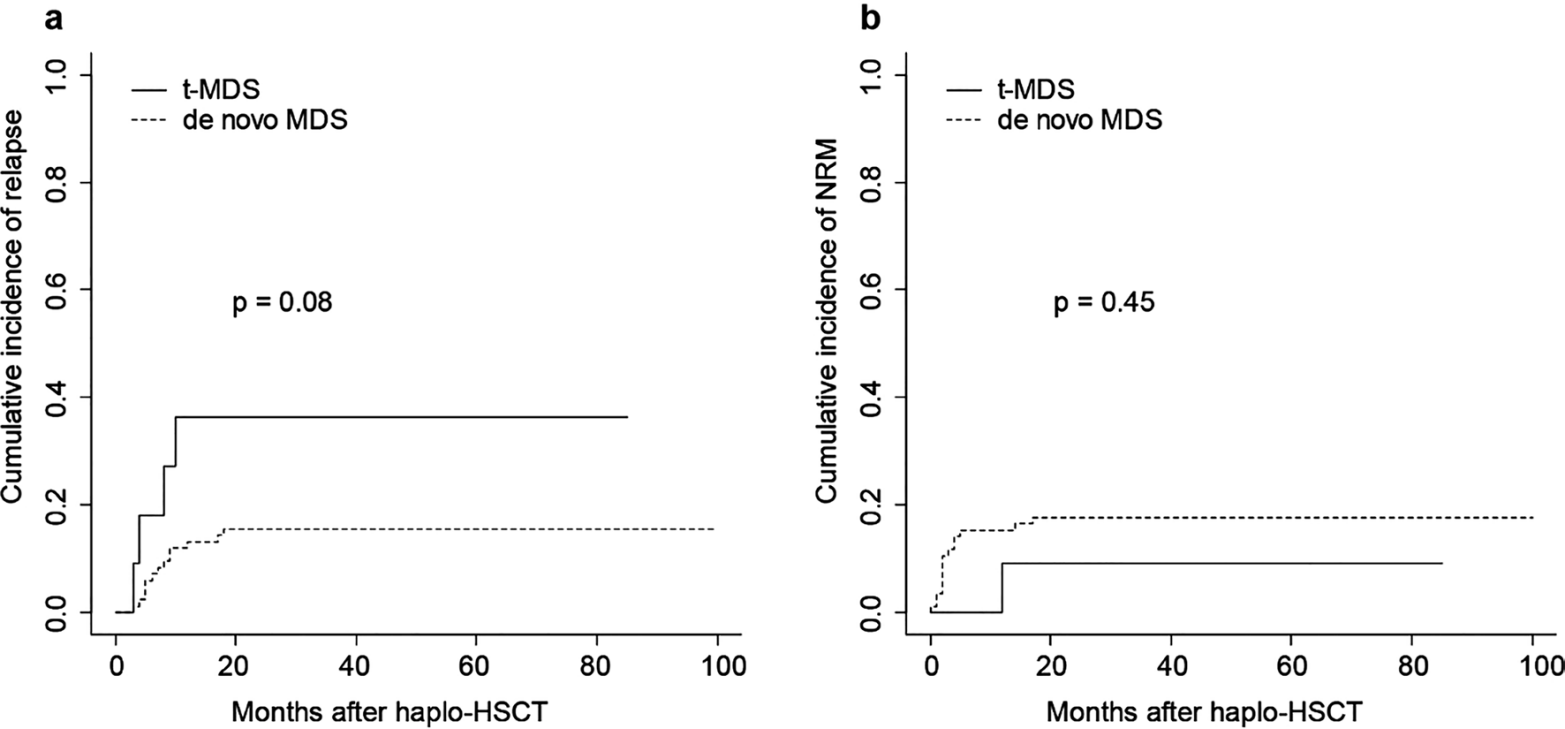
Cumulative incidence of relapse (**a**) and non-relapse mortality (**b**) after haplo-HSCT in patients with t-MDS or de novo MDS

Four patients (36.4%) in the t-MDS group and 13 (15.3%) in control cohort experienced relapse at a median of 7.3 months (range, 3.0–18.2 months) after haplo-HSCT. The relapse times of patients with t-MDS were 3.1, 3.6, 7.9, and 10.3 months after haplo-HSCT. The median times of relapse in the t-MDS and de novo MDS were 5.8 months (range, 3.1–10.3 months) and 7.3 months (range, 3–18.2 months), respectively. None of t-MDS patients experienced the recurrence of primary cancer. The 3-year cumulative incidence of relapse in the t-MDS group and control cohort was 36.4% and 15.5%, respectively (*P* = 0.08; Fig. 2a). In multivariable analysis, the incidence of relapse seemed to be comparable between patients with t-MDS and de novo MDS (Table 2). In univariate and multivariable analysis, high-risk cytogenetics was significantly correlated with a higher relapse rate [HR 2.758 (95% CI: 1.049–7.253), *P* = 0.040].

**Table 2:** Univariate analysis and multivariate analysis of overall survival, disease-free survival, relapse and non-relapse mortality

		Univariate analysis		Multivariate analysis	
	Variable	P value	HR (95% CI)	P value	HR (95% CI)
OS					
MDS type	t-MDS	0.990	1.008(0.301–3.371)		
	De novo MDS		1		
Age at haplo-HSCT	Age, < 35 years	0.049	0.235(0.055–0.996)	0.125	0.317(0.073–1.377)
	Age, ≥ 35 years		1		1
Platelet engraftment	Yes	0.000	0.069(0.028–0.174)	0.000	0.085(0.034–0.216)
	No		1		1
Graft type	PB	0.065	0.365(0.125–1.064)		
	BM + PB		1		
DFS					
MDS type	t-MDS	0.498	1.390(0.536–3.603)		
	De novo MDS		1		
Age at haplo-HSCT	Age, < 35 years	0.053	0.356(0.125–1.012)		
	Age, ≥ 35 years		1		
Platelet engraftment	Yes	0.000	0.096(0.040–0.228)	0.000	0.096(0.040–0.228)
	No		1		1
Relapse					
MDS type	t-MDS	0.127	2.394(0.780–7.352)		
	De novo MDS		1		
Cytogenetic risk	High risk	0.040	2.758(1.049–7.253)	0.040	2.758(1.049–7.253)
	Non-high risk		1		1
NRM					
MDS type	t-MDS	0.488	0.488(0.064–3.696)		
	De novo MDS		1		
Interval between MDS diagnosis and haplo-HSCT	Interval < 30 months	0.077	0.386(0.134–1.110)	0.058	5.321(0.945–29.961)
	Interval ≥ 30 months		1		1
Platelet engraftment	Yes	0.000	0.045(0.016–0.127)	0.000	0.012(0.002–0.066)
	No		1		1

*BM* Bone marrow, *CI* Confidence interval, *DFS* Disease-free survival, *haplo-HSCT* Haploidentical hematopoietic stem cell transplantation, *HR* Hazard ratio, *NRM* Non-relapse mortality, *OS* Overall survival, *PB* Peripheral blood

### NRM

One patient (9.1%) in the t-MDS group and 15 patients (17.7%) in the de novo MDS group died of NRM. The median time of NRM after haplo-HSCT was 2.4 months (range, 0.4–17 months). The NRM time of the patient in the t-MDS group was 11.5 months, and she died of infection. No t-MDS patients died of the recurrence of primary cancer. Patients in the control group died of infection (66.7%) and GVHD (13.3%). The cumulative incidences of NRM in the t-MDS group and control cohort were 9.1% and 17.6%, respectively (*P* = 0.45; Fig. 2b). In multivariable analysis for NRM, platelet engraftment was an independent significant factor [HR 0.012 (95% CI: 0.002–0.066), *P* = 0.000] (Table 2).

### DFS and OS

**Fig. 3:**
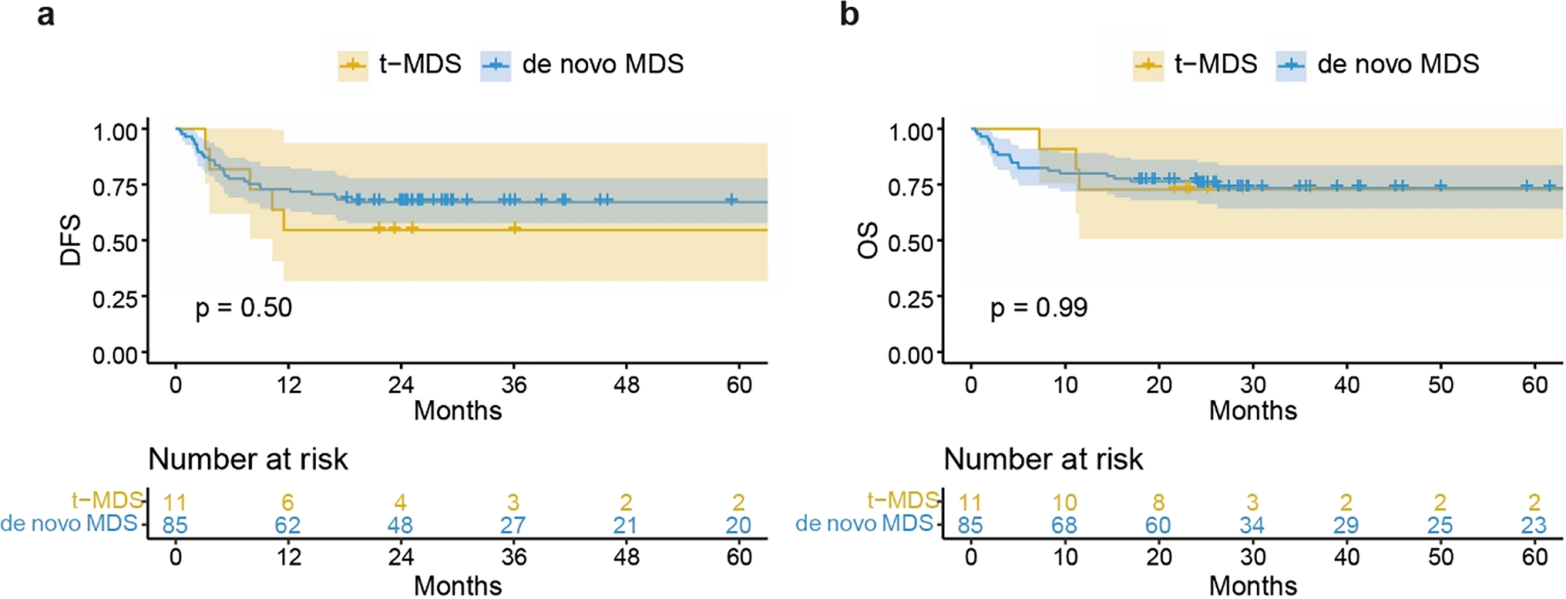
Disease-free survival (**a**) and overall survival (**b**) after haplo-HSCT between patients with t-MDS and patients with de novo MDS

The 3-year DFS for the t-MDS group and the de novo MDS group were 54.5% and 67.0%, respectively, *P* = 0.50 (Fig. 3a). The 3-year OS were 72.7% and 75.1%, respectively, *P* = 0.99 (Fig. 3b). In univariate analysis, the absence of platelet engraftment was significantly associated with higher DFS and OS [DFS: HR 0.096 (95% CI: 0.040–0.228), *P* = 0.000; OS: HR 0.069 (95% CI: 0.028–0.174), *P* = 0.000]. In multivariable analysis for DFS and OS, platelet engraftment remained a significant predictive variable [DFS: HR 0.096 (95% CI: 0.040–0.228), *P* = 0.000; OS: HR 0.085 (95% CI: 0.034–0.216), *P* = 0.000]. However, OS and DFS were not influenced by MDS type (t-MDS vs. de novo MDS) (Table 2).

## Discussion

To the best of our knowledge, this study is the first to systematically compare the clinical outcomes of haplo-HSCT on t-MDS and de novo MDS. Our study shows that the OS, DFS, relapse, and NRM of t-MDS after haplo-HSCT are comparable to those of de novo MDS.

In this study, we systematically analyzed the original case data of 11 t-MDS. Hematological malignancies were the most common primary cancer (54.5%). Kuendgen A et al. found that hematological malignancies (43%) were the most common primary tumor of t-MDS [4]. Our results are consistent with those of previous studies. In our study, the 3-year OS, DFS, relapse, and NRM rates in t-MDS patients were 72.7%, 54.5%, 36.4%, and 9.1%, respectively. In 2021, the CIBMTR reported that the OS, DFS, relapse, and NRM incidences in t-MDS after allo-HSCT were 26.9%, 19.4%, 46.2%, and 33.7%, respectively [17]. Compared with the results of the CIBMTR, our clinical outcomes of t-MDS after haplo-HSCT are encouraging. Several possible explanations might account for these findings. First, the median age of our group was younger than that of the CIBMTR (48 years vs. 58.6 years). In previous studies of t‐MNs from CIBMTR and EBMT, older age was identified as an independent risk factor for post‐transplant survival [8, 18]. Second, the data of CIBMTR of allo-HSCT were between 2000 and 2014, during an early era of transplantation, while the median year of our data was 2019 (range, 2015–2021). Third, patients in our study merely received haplo-HSCT, whereas the CIBMTR study concentrated on HLA identical sibling and unrelated donor settings. Survival was promising owing to the stronger graft-versus-leukemia effects of haploidentical HSCT [24]. Fourth, our patients all received MAC, in contrast to the study of CIBMTR in which 51% of patients received reduced-intensity conditioning (RIC). A previous study of patients with MDS eligible for either the MAC or RIC regimens and long-term follow-up demonstrated a survival advantage for patients who received MAC [25]. Fifth, our center has uniform treatment schemes including conditioning regimens and GVHD prophylaxis, whereas the CIBMTR study analyzed data from multiple centers with heterogeneous patient groups and treatment methods.

In this study, the clinical outcomes including the rates of relapse, OS, DFS, and NRM after haplo-HSCT for patients with t-MDS were comparable to those with de novo MDS. However, relapse seems to be a major challenge of haplo-HSCT for patients with t-MDS, although the *P* value in univariate and multivariate analyses did not indicate a significant difference. The trend of a high relapse rate may be related to the overall small number of patients in our retrospective study, so it needs to be further confirmed by prospective large samples of studies. It is might associated with high-risk cytogenetics [5, 18], because that the proportion of patients with high-risk cytogenetic abnormalities was higher in t-MDS (45.5%) than in de novo MDS (18.8%) (*P* = 0.04). Ibrahim Aldoss et al. found comparable outcomes between t-MDS and de novo MDS after HLA identical sibling and unrelated donor transplants (OS, 49.9% vs. 53.9%, *P* = 0.61; relapse-free survival, 47.2% vs. 49.5%, *P* = 0.68) [5]. However, we found that the OS (72.7% vs. 75.1%; *P* = 0.99) and DFS (54.5% vs. 67.0%; *P* = 0.50) of t-MDS and de novo MDS in our study seem more encouraging than that in previous studies, which might because of that the age of patients were young and all received haplo-HSCT with a myeloablative conditioning regimen in our study. Therefore, haplo-HSCT is an optimum and curative therapeutic option for patients with t-MDS. In our study, platelet engraftment was an independent significant factor for OS, DFS and NRM. Our previous study indicated that primary prolonged isolated thrombocytopenia was significantly associated with inferior overall survival and higher TRM, which is consistent with this study [26].

Limitations still exist in our study. First, our study is a single-center and retrospective study, which might lead to selection bias. Second, the number of t-MDS cases in our center was relatively small, which might lead to statistical bias. Third, because the primary cancer of some t-MDS patients was not diagnosed and treated in our center, we lack some data on the details of the initial therapy of the primary cancer. Fourth, our data began in 2015, while next-generation sequencing (NGS) was carried out in our center in 2018, so part of the data lacked the results of NGS. Therefore, we expect more multicenter and prospective studies to confirm our conclusions, and we also hope that research will further prove the biological characteristics of t-MDS in future.

In conclusion, our study first demonstrated that t-MDS has comparable outcomes to de novo MDS after haplo-HSCT. Therefore, haplo-HSCT is one of the feasible options for t-MDS patients if there is no HLA-matched sibling. Additionally, large, well-designed, prospective randomized trials are needed to further confirm these conclusions.

## Data Availability

The datasets generated during and/or analyzed during the current study are available from the corresponding author on reasonable request.
